# Post-Acute COVID-19 Symptoms Clusters and Work Status

**DOI:** 10.3390/occuphealth1030031

**Published:** 2026-07-15

**Authors:** Aoyjai Prapanjaroensin Montgomery, Nathaniel Erdmann, Wanda Hall, Juan Pablo Pilco, Emily B. Levitan

**Affiliations:** 1 Department of Family, Community and Health Systems, School of Nursing, University of Alabama at Birmingham, Birmingham, AL 35294, USA; 2 Division of Infectious Diseases, Department of Medicine, School of Medicine, University of Alabama at Birmingham, Birmingham, AL 35294, USA; 3 Center for Clinical and Translational Science (CCTS), University of Alabama at Birmingham, Birmingham, AL 35294, USA; 4 Clinical and Diagnostic Sciences Department, School of Health Professions, University of Alabama at Birmingham, Birmingham, AL 35294, USA; 5 Department of Epidemiology, School of Public Health, University of Alabama at Birmingham, Birmingham, AL 35294, USA

**Keywords:** COVID-19, post-acute COVID-19 syndrome, employment status, long COVID brain fog, return to work

## Abstract

Long COVID affects an estimated 10–20% of SARS-CoV-2 survivors with heterogeneous, multi-system symptoms persisting for months to years, contributing to significant workforce disruption and reduced functional capacity. This exploratory study identified symptom clusters following SARS-CoV-2 infection, examined associated personal and clinical characteristics, and assessed work status across clusters. Among 273 individuals from a single clinical site, we used baseline surveys to capture long COVID symptoms and 2-week follow-up surveys to assess work status, and applied K-means clustering to identify symptom groupings. Three preliminary clusters emerged: Cluster 1 (“brain fog”), characterized by fatigue (96%), difficulty remembering (97%), and difficulty concentrating (94%); Cluster 2 (“undifferentiated”), with low prevalence across all symptoms; and Cluster 3 (“persistent acute”), marked by cough (92%), congestion (80%), headaches (78%), and body aches (78%). Notably, among Cluster 1 (“brain fog”) respondents, 44% reported not working, and approximately half attributed their work absence to COVID-related symptoms. These hypothesis-generating findings suggest that neurocognitive symptom clusters may be associated with greater work absence in some individuals, though the small number of respondents limits firm conclusions. The findings suggest that targeted occupational rehabilitation strategies may warrant further investigation as potential strategies for individuals experiencing post-infectious cognitive impairment following SARS-CoV-2 infection.

## 1. Introduction

Long COVID serves as a salient example of a post-infectious syndrome, illustrating the broader phenomenon in which a subset of individuals experience prolonged, often debilitating symptoms following the resolution of an acute infection. Although a majority of individuals infected with SARS-CoV-2 recover in the short term, 10–20% of people (estimated 10–20 million cases worldwide) exhibit persistent symptoms, referred to as “long COVID” or post-acute sequelae of COVID-19 [1-3]. According to the National Academies of Sciences, Engineering, and Medicine, long COVID is classified as an infection-associated chronic condition that develops following a SARS-CoV-2 infection and persists for at least three months [4]. It may present as a continuous, relapsing–remitting, or progressive disease affecting one or more organ systems [4]. Four classifications of post-COVID symptoms have been proposed: (A) transition phase (symptoms onset to 5 weeks), (B) acute post-COVID symptoms (5–12 weeks), (C) long COVID symptoms (12–24 weeks), and (D) persistent post-COVID symptoms (symptoms lasting longer than 24 weeks) [5]. Long COVID syndromes encompass a wide range of symptoms across systems including neurocognitive (dizziness, loss of attention, brain fog, confusion), autonomic (tachycardia, chest pain, palpitations), gastrointestinal (abdominal pain, diarrhea, vomiting), respiratory (cough, sore throat, fatigue, dyspnea), musculoskeletal post-COVID (myalgias, arthralgias), and psychological-related post-COVID (depression, insomnia, post-traumatic stress disorder, anxiety) symptoms [5]. Prior studies have suggested multiple subtypes of long COVID; however, methods for identifying subgroups are not established, and the identified subtypes of long COVID are not consistent across study populations [6-9].

Millions are now dealing with various manifestations of long COVID, and the symptoms can impact lifestyle and work. A group of researchers from the United Kingdom and the United States conducted an international cohort study (3762 participants from 56 countries) on how prolonged COVID-19 symptoms impact daily functioning and found 45% of participants required a reduced work schedule and an additional 22% were not working due to COVID-19 symptoms [10]. The United States Census Bureau collaborated with multiple federal agencies to survey the social and economic effects on American households during the pandemic [11]. As of November 2022, over 27 million households reported the loss of employment income, while 1.5 million were laid off or furloughed due to the coronavirus pandemic, and 1.6 million were sick or caring for someone with COVID-19 [12]. It is important to understand the long-term physical, psychological, and cognitive consequences of long COVID from a population perspective and how these symptoms affect occupational functioning. The purposes of this study were to (1) explore symptom clusters following SARS-CoV-2 infection, (2) characterize the personal (age, gender, race) and clinical (hospitalization, length of stay, and COVID-19 diagnosis date) characteristics based on exploratory symptom clusters, and (3) examine work status and reason for not working based on exploratory symptom clusters.

## 2. Methods and Participants

### 2.1. Study Design and Setting

This is a secondary data analysis of a parent study called the ‘COVID-19 Enterprise Biorepository and Data Registry,’ which was designed to support the systematic recruitment of individuals with acute COVID-19 and the collection of specimens and clinical data with longitudinal follow-up. The project setting was an urban academic health science center located in the southeastern United States and began in April 2020 as the first cases of COVID-19 were being reported in the region. This academic health center is the 3rd largest public hospital in the United States. The hospital has 1207 licensed beds and sees an average of 55,000 admissions per year and 6000 ambulatory visits per day, and since 2002 is a five-time American Nurse Credentialing Center (ANCC) Magnet-designated facility. From April 2020 to February 2022, there were 96,680 total admissions, of which 7467 (8%) had a COVID-19 diagnosis.

### 2.2. Sample

Participants treated for COVID-19 within the health system were eligible to participate. Inclusion criteria included (1) age 18 years or older, (2) being clinically diagnosed with COVID-19, (3) treated at the urban academic health science center located in the southeastern United States, and (4) provided verbal consent for collection of biospecimens, use of medical records for research, and recontact for further research. Although participants at all levels of COVID-19 severity were eligible, recruitment efforts focused on individuals hospitalized for COVID-19 and those who were treated with monoclonal antibodies for prevention of hospitalization. After the acute SARS-CoV-2 infection and, for those hospitalized, discharge from the hospital, participants were invited to return for clinic visits which included electronic surveys on symptoms and other characteristics completed through REDCap^®^ software (version 17.2.1). This first post-acute research visit was considered the baseline for these analyses and included COVID-19 symptoms survey. Participants were invited to complete a follow-up survey approximately 2 weeks later which included the work status and the main reason for not working survey items. This study uses the term ‘post-acute COVID-19’ broadly to refer to the period following resolution of acute SARS-CoV-2 infection, without applying a strict temporal cutoff to distinguish post-acute from long COVID. Participants were surveyed at their first post-acute research visit, which occurred at variable time points after acute infection.

Participants’ demographic and clinical characteristics were obtained from electronic health records. A total of 547 participants responded to the baseline survey. To examine symptom clusters, we required that all participants complete all symptom survey questions (excluding 140 participants with missing values, 30 who responded “prefer not to report” for one or more symptoms, and 104 who responded that they were not sure if they had one or more symptoms). The final sample was 273 participants. Comparisons of demographic and clinical characteristics between the 273 included and 274 excluded participants revealed no statistically significant differences after correction for multiple comparisons (Benjamini–Hochberg false discovery rate correction; all q > 0.05). Effect sizes were small across all variables (Supplementary Table S1), indicating limited systematic selection bias by measured characteristics in the final analytic sample.

### 2.3. Data Collection

Data included participants’ demographics as recorded in electronic medical records, with the COVID-19 symptoms survey including items adapted from existing work on COVID-19 and other infectious diseases, and the work status and the main reason for not working questions based on items used in the US Census Bureau Household Pulse surveys. Details of the instruments are below.

#### 2.3.1. Electronic Medical Records

Participants’ demographics including age, gender, race and clinical characteristics including COVID-19 diagnosis date, if they were hospitalized, and if so, the hospital length of stay were retrieved from electronic medical records.

#### 2.3.2. Post-Acute COVID-19 Symptoms Survey

The post-acute COVID-19 symptoms survey consisted of 24 questions drawn from various studies and survey instruments. Three questions that asked if a participant feels back to their usual health, has fatigue or extreme tiredness, and has body aches were from the Centers for Disease Control and Prevention (CDC) Morbidity and Mortality Weekly Report (MMWR) [13]. Fourteen questions that asked if a participant still has the following symptoms, fever (temp > 100.4 F), felt feverish, chills, muscle aches, runny nose or congestion, sore throat, cough, shortness of breath or difficulty breathing (dyspnea), nausea or vomiting, headaches, abdominal pain, loss of taste, loss of smell, and diarrhea were from the MMWR and the Multicenter AIDS Cohort Study/Women’s Interagency HIV Study Combined Cohort Study (MWCCS) COVID-19 symptom survey [13,14]. Three questions on confusion, difficulty concentrating, and difficulty remembering things were adapted from a large general population study [15]. A single question on physical activity was adapted from the Patient Reported Outcome Measurement Information System (PROMIS) question bank [16]. The two-item Patient Health Questionnaire (PHQ-2) was included to measure depression [17]. Lastly, a single item on anxiety asking “How much do you agree with the following statement? My anxiety levels are higher than normal” was included.

#### 2.3.3. Work Status at Post-Acute Infection

Two questions from the United States Census Bureau survey on measuring the experience of how the coronavirus pandemic impacts households from social and economic perspective were included in the 2-week follow-up survey. The first question was “In the last 7 days, did you do ANY work for either pay or profit?” Four possible responses were ‘Yes’, ‘No’, ‘Don’t know/Not sure’, and ‘Prefer not to answer’. Then, the follow-up question for those who responded as not working was “what is your main reason for not working for pay or profit?” with 12 possible responses: (1) I did not want to be employed at this time, (2) I am/was sick with coronavirus symptoms or caring for someone who was sick with coronavirus symptoms (including long-term effects of coronavirus), (3) I am/was caring for children not in school or daycare, (4) I am/was caring for an elderly person, (5) I was concerned about getting or spreading the coronavirus, (6) I am/was sick or disabled (not coronavirus related), (7) I am retired, (8) I am/was laid off or furloughed due to coronavirus pandemic, (9) My employer closed temporarily due to the coronavirus pandemic, (10) My employer went out of business due to the coronavirus pandemic, (11) I do/did not have transportation to work, and (12) Other reason, please specify [11].

### 2.4. Statistical Analysis

Participant demographic and health information from the electronic health record, post-acute COVID-19 symptoms from the baseline survey and the work status and the main reason for not working from the 2-week follow-up survey were merged by medical record number. Descriptive statistics were tabulated for overall participant personal characteristics (including age, gender, race), clinical characteristics (including hospitalization and length of stay), COVID-19 symptom questions, and the work status and the main reason for not working. We used K-means clustering to identify groups of participants based on the 24 self-reported, potentially COVID-19-related symptoms using the ‘cluster’ and ‘factoextra’ packages in R [18,19]. K-means clustering was selected because it is computationally efficient, produces easily interpretable clusters, and has been applied extensively in COVID-19 and long COVID symptom clustering studies [6,7,9]. The 24 symptom variables entered into the K-means clustering included two types: (a) binary indicators (yes/no presence of individual symptoms such as cough, fever, fatigue, confusion, and others), coded as 0 = absent and 1 = present, with all yes/no items recoded to a consistent direction prior to analysis; and (b) ordinal items including physical activity (5 response levels), the two PHQ-2 depression items (4 response levels each), and the anxiety item (5-point Likert scale), which were treated as numeric integers. All 24 variables were standardized (mean = 0, SD = 1) using the scale() function in R prior to clustering (set.seed(123)), ensuring that variables with differing response ranges contributed equally to the distance calculations. The study is explicitly exploratory in nature, and all findings are interpreted accordingly throughout. K-means clustering is an unsupervised learning model that groups participants into clusters based on the sum of the distance between data points, so that individuals with similar sets of symptoms are in the same category [20]. This method is based on calculating the within-cluster sum of squared errors for different numbers of clusters [20]. To determine the optimal number of clusters, we used the elbow method in conjunction with examination of gap statistics and consideration of parsimony and clinical interpretability in the context of the modest sample size. To further assess cluster robustness, average silhouette widths were calculated. Next, the descriptive statistics of the personal and clinical characteristics, 24 COVID-19 symptoms, the work status, and the reason for not working were tabulated based on assigned cluster. The date of COVID-19 diagnosis was plotted by month based on assigned k cluster. All data merging, cleaning, and analyses were conducted using R environments for statistical computing (version 2022.07.1) [21].

## 3. Results

### 3.1. Demographics and Descriptive Statistics

Table 1 shows the characteristics of the 273 participants with history of COVID-19. The average age of participants at COVID-19 diagnosis was 52 years. The majority of participants were female (51%), White (58%), and did not require admission to the hospital during their acute COVID-19 infection (63%). For those who were admitted to the hospital (*n* = 58, 21%), the average stay was 11 days.

Table 2 shows the frequency of post-acute COVID symptoms reported by participants. The five most frequent symptoms were fatigue or extreme tiredness (*n* = 155, 57%), body aches (*n* = 119, 44%), difficulty remembering things (*n* = 116, 43%), headaches (*n* = 107, 39%), and difficulty concentrating (*n* = 106, 39%). The largest proportion of participants reported being completely able to carry out everyday physical activity (*n* = 111, 41%), and the majority were not bothered by a lack of pleasure in doing things (*n* = 167, 61%), and were not feeling down, depressed, or hopeless (*n* = 191, 70%). Approximately 44% of participants (*n* = 119) responded that their anxiety levels were not higher than normal, while 39.6% of participants (*n* = 108) responded that they were.

Table 3 shows the frequency and percentage of participants who responded to the work status (working for either pay or profit) and the main reasons for not working. Only 127 participants responded to the two-week follow-up survey. We conducted comparisons between participants who completed the follow-up survey and those who did not on key demographic characteristics. Results indicated no statistically significant differences in age (Welch *t*-test, *p* = 0.060), gender (*p* = 0.583, Cramér’s *V* = 0.036), or race (*p* = 0.280, Cramér’s *V* = 0.163). Effect sizes were small across all comparisons, suggesting that the analytic sample was not systematically different from non-completers on these key characteristics. Approximately 59% (*n* = 75) reported that they were working for either pay or profit, while 52 participants (41%) reported that they were not working. Those 52 participants responded to the main reason for not working prompt. The five reported reasons for not working were retirement (*n* = 18, 6.6%), other reasons (*n* = 13, 4.8%), sickness or disability (not coronavirus related) (*n* = 8, 2.9%), sickness related to coronavirus symptoms (*n* = 7, 2.6%), and reduction in business due to the coronavirus pandemic (*n* = 3, 1.1%).

### 3.2. Post-Acute COVID-19 Symptom Clusters of Confirmed SARS-CoV-2 Infection

**Figure 1.**
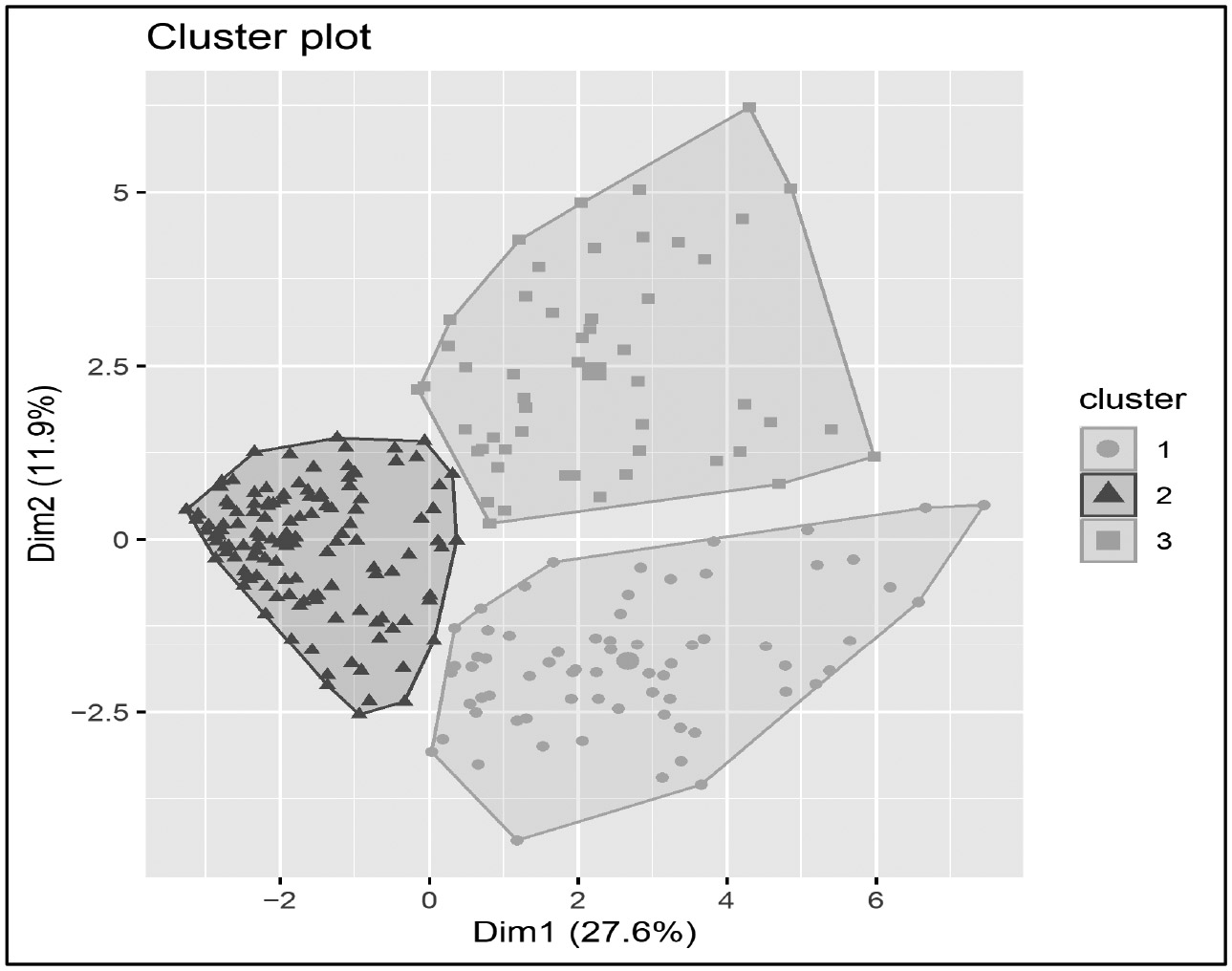
K-means clustering solution visualized using principal component analysis (PCA). Axes represent the first two principal components (Dim1: 27.6% of variance; Dim2: 11.9% of variance). Each point represents one participant; patterns indicate cluster assignment (Cluster 1: ‘brain fog’, *n* = 68; Cluster 2: ‘undifferentiated’, *n* = 154; Cluster 3: ‘persistent acute’, *n* = 51).

The elbow method with a minimum of 25 samples in each cluster recommended a three-cluster solution. The elbow plot used to guide cluster selection is presented in Supplementary Figure S1. Cluster 1, 2, and 3 included 68, 154, and 51 participants, respectively. The clustering explained 28.1% of the total variance in the data set (between cluster sum of squares/total cluster sum of squares; BSS/TSS). To evaluate the optimal number of clusters, K-means solutions with k = 2, 3, and 4 were compared. The two-cluster solution (BSS/TSS = 20.2%) produced substantial cluster overlaps on the first two principal components, limiting clinical interpretability. The four-cluster solution (BSS/TSS = 32.6%) offered only a marginal improvement over the three-cluster solution (BSS/TSS = 28.1%), without proportionate gain in clinical distinctiveness. The three-cluster solution was therefore selected as the most parsimonious and interpretable. We additionally examined the gap statistic to further evaluate the optimal number of clusters. The gap statistic continued to increase with k and suggested a larger number of clusters (k = 10 under the firstmax criterion; set.seed(123), K.max = 10, B = 50). Given the small sample size, a ten-cluster solution was not clinically interpretable or feasible. The three-cluster solution was therefore retained as the most parsimonious and clinically meaningful exploratory model, selected on the basis of the elbow method, BSS/TSS comparisons across k = 2, 3, and 4, and clinical interpretability rather than as a statistically definitive solution. To further assess cluster robustness, average silhouette widths were calculated for k = 2, 3, and 4. The three-cluster solution yielded the highest average silhouette width (k = 3: 0.25; k = 2: 0.24; k = 4: 0.21), providing modest support for the three-cluster solution relative to alternatives. At the cluster level, Cluster 2 (‘undifferentiated’) showed the strongest separation (silhouette width = 0.42), while Cluster 1 (‘brain fog’) showed weak separation (0.08) and Cluster 3 (‘persistent acute’) showed a near-zero silhouette width (−0.02), indicating limited distinctiveness from the other clusters. The silhouette plot is presented in Supplementary Figure S2. Figure 1 shows a graphic for K-means clustering with three clusters. As is inherent to K-means clustering, each participant was assigned to exactly one cluster based on minimum Euclidean distance to the cluster centroid.

Next, post-acute COVID-19 symptoms were tabulated based on the assigned clusters (Table 2). Cluster 1 showed higher percentages of participants with difficulty remembering things (97%), fatigue or extreme tiredness (96%), difficulty concentrating (94%), shortness of breath or difficulty breathing (dyspnea) (78%), confusion (72%), and abdominal pain (25%). Therefore, Cluster 1 is labeled the ‘brain fog’ cluster. Cluster 2 showed low prevalence of all symptoms and is labeled the ‘undifferentiated’ cluster. Lastly, Cluster 3 showed higher percentages of participants with cough (92%), runny nose or congestion (80%), headaches (78%), body aches (78%), muscle aches (73%), loss of taste (59%), loss of smell (59%), chills (47%), diarrhea (41%), felt feverish (39%), sore throat (35%), nausea or vomiting (31%), not feel back to usual health (28%), and fever (18%); Cluster 3 is labeled the ‘persistent acute’ cluster. The ‘persistent acute’ cluster label describes a symptom pattern consistent with ongoing or slowly resolving acute COVID-19 symptoms. The term is used descriptively rather than as a formal clinical classification.

### 3.3. Demographics and Pattern of COVID-19 Diagnosis Date Based on Symptom Clusters

**Figure 2.**
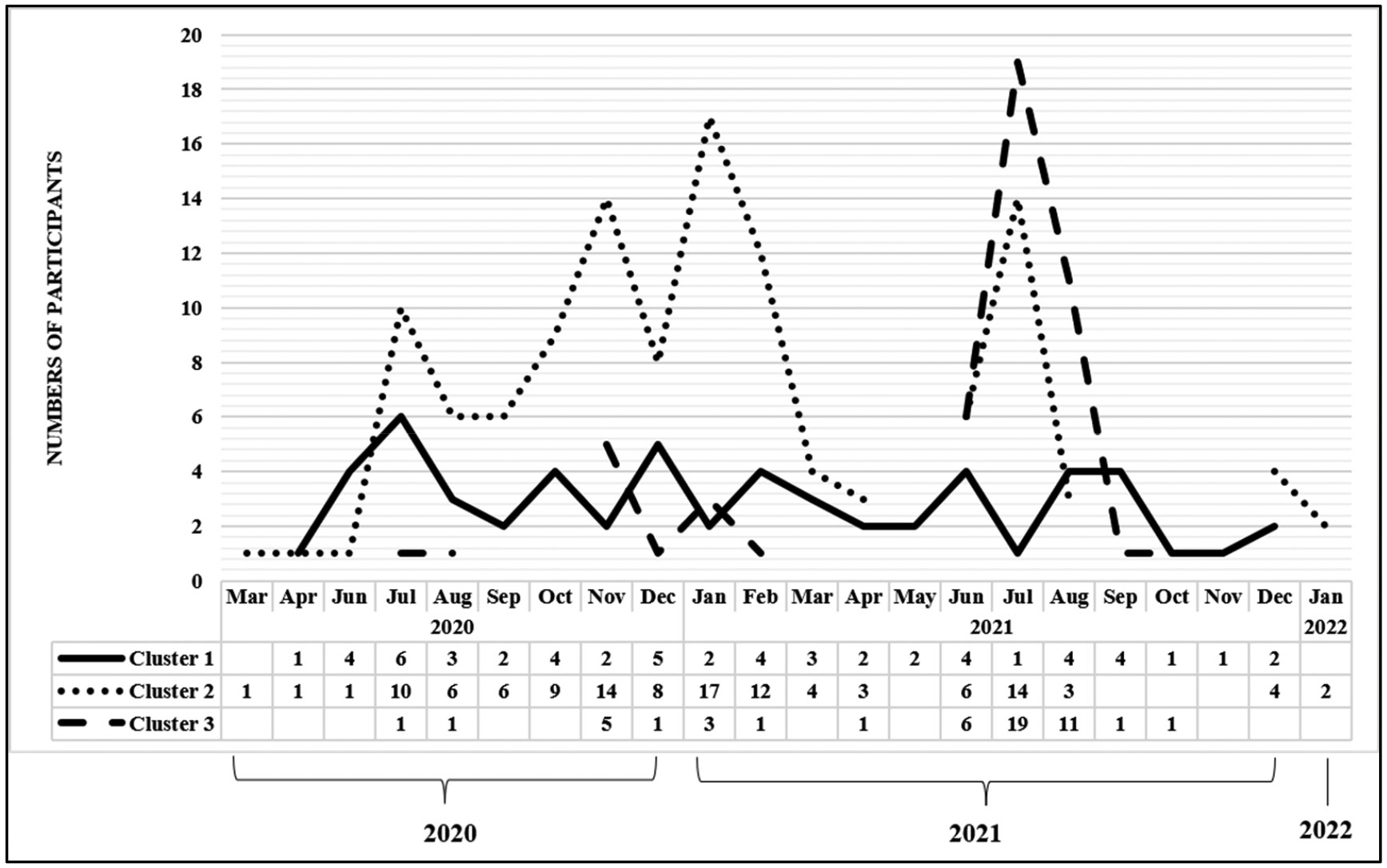
Dates of COVID-19 diagnosis based on the assigned clusters.

The majority of participants in all clusters were female and White (Table 1). Participants in the ‘brain fog’ cluster were older (mean 57 years) compared to the ‘undifferentiated’ cluster (mean 55 years), and ‘persistent acute’ cluster (mean 46 years). Even though only 16% of participants in the ‘brain fog’ cluster were hospitalized, among those who were hospitalized the average length of stay was longer (13 days) than participants from other clusters (average of 10 days for both ‘undifferentiated’ and ‘persistent acute’ clusters). Figure 2 shows dates of COVID-19 diagnosis based on the assigned clusters. Participants in the ‘brain fog’ cluster were distributed across the full duration of the COVID-19 pandemic. Participants from ‘undifferentiated’ were more likely from the early COVID-19 through July 2021 while participants in ‘persistent acute’ were more likely from later in the COVID-19 pandemic (June to August 2021).

### 3.4. Work Status and the Main Reason for Not Working Based on Symptoms Clusters

Based on the symptom clusters (baseline survey), work status and the main reasons for not working (follow-up survey) were categorized and shown in Table 3. Approximately 44% of participants (*n* = 14) in ‘brain fog’ were not working for either pay or profit and 50% of these participants (*n* = 7) were because they were sick with coronavirus symptoms. Smaller percentages of participants in the ‘undifferentiated’ and ‘persistent acute’ clusters (39% and 43%, respectively) were not working, and the main reason was elective retirement (38% and 67%, respectively).

## 4. Discussion

Using self-reported post-acute symptoms among individuals with a history of SARS-CoV-2 infection, we preliminarily identified three clusters. (1) The ‘brain fog’ cluster consists of participants with severe post-acute symptoms, including poor cognitive performance, which in this exploratory analysis appeared to be associated with their work status. Individuals in this cluster were older, and those who were hospitalized stayed in hospitals longer than those in other clusters. (2) The ‘undifferentiated’ cluster consists of participants with no dominant symptoms. These individuals were more likely to have had initial COVID-19 infection from early in the pandemic through July 2021. (3) The ‘persistent acute’ cluster consists of participants with mild-to-moderate symptoms. These individuals were more likely from later in COVID-19 pandemic (June to August 2021) with a shorter time between illness and survey completion.

Overall, we found that the most common post-acute symptoms were fatigue or extreme tiredness, body aches, and difficulty remembering things. These findings are congruent with a study from 2021 assessing 2550 participants [22]. Ziauddeen and colleagues reported that exhaustion was an initial symptom of acute COVID-19 that persisted as a long COVID symptom, while cognitive dysfunction was a common long COVID symptom that developed after the acute phase [22]. The temporal concentration of the ‘persistent acute’ cluster during June–August 2021 coincides with the predominance of the SARS-CoV-2 Delta variant in the United States during this period. Different SARS-CoV-2 variants have been associated with distinct symptom profiles and long-term sequelae; pre-Delta variants were more frequently linked to neurological and olfactory post-acute sequelae, while the Delta variant was associated with more prominent upper and lower respiratory symptoms [23], potentially contributing to the respiratory-predominant symptom pattern observed in the ‘persistent acute’ cluster. Our finding of three clusters is both consistent with and divergent from previous work. Prior work has relied upon different patient populations and clustering techniques (e.g., K-medoids, K-means, unsupervised agglomerative hierarchical, and agglomerative hierarchical) [6-8,22,24]. Danesh and colleagues conducted a study among 441 outpatients at COVID-19 recovery clinics in Texas and found two clusters (neuropsychiatric and pulmonary clusters) [6]. Reese and colleagues analyzed the NIH N3C and RECOVER electronic health record program data and found six clusters of PASC symptoms [6,8]. The RECOVER adult cohort which assessed participant-reported symptoms identified four clusters, three of which were classified by high prevalence of fatigue and brain fog (84–100%) [9].

Even though some studies found three symptom clusters similar to the current study, the symptoms were slightly different in the clusters [7,22,24]. Frontera and team included 242 patients hospitalized with COVID-19 and reported three clusters: the first with headache, cluster 2 with high levels of anxiety and depression, and cluster 3 with shortness of breath, and cognitive symptoms [7]. Ziauddeen and colleagues used an online social media survey (n = 2550) and reported that two main symptom clusters exhibited mainly chest, cognitive symptoms, and exhaustion, and the minority cluster exhibited multi-system symptoms that had persisted from the initial symptoms [22]. Furthermore, a group of researchers in Spain conducted a multicenter study among 2000 participants [24] and found three clusters with slightly different symptoms compared to our findings. Clusters 1 and 2 exhibited respiratory symptoms, limited daily living activities, higher anxiety and depressive levels, and worse sleep quality, while cluster 3 exhibited low prevalence of all symptoms [24]. Differences in findings across studies likely reflect variation in study populations, symptom questionnaires, and clustering methodologies. Our population was mainly from Birmingham, Alabama, in the United States, while Ziauddeen’s study was among residents of the United Kingdom, and Fernández-de-las-Peñas’s study was among residents of Spain. Items in questionnaires from Ziauddeen’s study were not exhibited in their paper. Fernández-de-las-Peñas and colleagues stated that they predefined a list of post-COVID symptoms for their instrument [24]. In the United States RECOVER adult cohort, only participants who met a long COVID symptom threshold were included in the symptom cluster analysis [9].

The United States Census Bureau reported that in November 2022, approximately 44% of Alabama residents (1,691,024 out of 3,834,458 households) were not employed in the last 7 days with 2.7% (105,363 Alabama residents) because they were sick or caring for someone with COVID-19; however, the proportion that were not working because of their own illness (separate from caregiving) was not reported [12]. Our findings found only 3% of participants reported that they did not work because of being sick with coronavirus symptoms and many participants were retired. This may in part reflect retired persons having more time to attend study visits, which were conducted during usual business hours. In contrast, Ziauddeen and colleagues reported that 37% of 2550 participants reported loss of income due to illness [22]. Our findings show all participants who reported that they did not work because of coronavirus symptoms were in the ‘brain fog’ cluster (with fatigue and cognitive impairments). A previous study stated that COVID-19 may impair cognition due to the systemic inflammation that persists after acute illness and affects quality of life, which is related to working life [25]. A systematic review study on COVID-19-associated cognitive impairment reported that 13 of 22 studies identified a pattern of impairment in processing speed, inattention, or executive dysfunction. These cognitive deficits may directly affect work performance and contribute to difficulty returning to work following COVID-19 infection [26].

### 4.1. Limitations

We note several limitations to this study. The implications of the presented findings are limited by the sample size and single-site study design; therefore, a larger population will be needed to confirm and expand upon these findings. The study sample was recruited from a single tertiary academic center and was enriched for more severe acute COVID-19 presentations, including hospitalized patients and those treated with monoclonal antibodies for prevention of hospitalization. This limits the generalizability of our findings to the broader working population with long COVID, in whom symptom severity, relative cluster frequency, and cluster composition may differ substantially. Employment data were available for only 127 of 273 participants (46.5%), substantially limiting ability to draw conclusions. The relatively high mean age of the sample (~52 years) and the high proportion of retirees among non-working participants limit the ability to attribute work absence specifically to long COVID symptoms. All employment-related findings should be interpreted as exploratory and hypothesis-generating. Further, the response rate to offered surveys may be a source of bias. We measured employment at a single time point and did not assess ability to return to work as long COVID symptoms evolved. This study relied exclusively on self-reported symptom data, which may not fully capture the breadth of long COVID’s impact on occupational functioning. Standard K-means clustering relies on Euclidean distance and is most appropriate for continuous variables. Although all variables were standardized prior to clustering, the symptom data included binary indicators and ordinal items, and standardization does not fully resolve the limitations of applying Euclidean distance to such data. Alternative methods designed for categorical or mixed-type data, such as K-modes, K-prototypes, Gower distance-based clustering, or Latent Class Analysis, may yield different cluster structures. This should be considered when interpreting the identified clusters, which are best understood as preliminary and exploratory groupings rather than validated clinical phenotypes. Average silhouette widths were calculated to assess cluster robustness. The overall average silhouette width for the three-cluster solution was 0.25, indicating weak but non-trivial cluster separation. At the cluster level, the ‘undifferentiated’ cluster showed reasonable separation (0.42), while the ‘brain fog’ (0.08) and ‘persistent acute’ (−0.02) clusters showed limited distinctiveness. These findings underscore the importance of interpreting the identified clusters as preliminary and exploratory symptom groupings rather than as statistically validated or discrete clinical phenotypes. Importantly, research has demonstrated that even mild acute SARS-CoV-2 infection and infections with no or few symptoms can lead to objective, measurable physiological alterations including reduced pulmonary diffusion capacity, autonomic nervous system dysfunction, microstructural brain changes on MRI, and impaired exercise tolerance that may directly impair work capacity independent of, or in excess of, subjective symptom reporting. Future research with longitudinal designs that follow individuals over time, and incorporate measures of symptom severity and healthcare provider assessments (e.g., magnetic resonance imaging of the brain, chest X-rays, and pulmonary function tests) would provide additional insight into the subtypes of long COVID syndromes and their relationships with the ability to work. The baseline survey was completed at variable time points following acute SARS-CoV-2 infection, without standardization of the assessment interval. This introduces heterogeneity into the symptom reports and may partially account for the identified cluster patterns, particularly the ‘persistent acute’ cluster, whose symptom profile may reflect a shorter time since acute illness rather than a distinct chronic phenotype.

### 4.2. Implications for Occupational Health Practice

Long COVID symptoms pose a significant challenge for occupational health, particularly for individuals experiencing severe symptoms related to fatigue and cognitive impairment, which the prior literature suggests can hinder work performance and employability. This study highlights the ‘brain fog’ cluster as a potentially key concern, suggesting that individuals with these symptoms may require targeted support to successfully reintegrate into the workforce. Developing workplace accommodations, rehabilitation programs, and return-to-work strategies tailored to employees with cognitive and physical impairments may help individuals with long COVID successfully return to work. Practical accommodations for workers with cognitive long COVID may include graduated return-to-work programs with progressively increasing demands, simplified task instructions, reduced multitasking requirements, flexible scheduling, and structured workflows to support working memory. Occupational screening using validated cognitive tools (e.g., PROMIS Cognitive Function) at post-COVID occupational health assessments may help identify workers requiring targeted support.

Further research is urgently needed to identify effective interventions for managing the physical, mental, and emotional symptoms that impact work capacity among persons with long COVID and other post-infectious syndromes. Implementing strategies to mitigate severe long COVID symptoms—such as workplace flexibility, occupational therapy, and mental health support—can improve individual recovery while benefiting overall workforce productivity. Addressing these challenges through evidence-based occupational health policies will be crucial in promoting long-term workplace sustainability and employee well-being. For example, findings from a small-scale pilot randomized controlled trial [27] suggest that Constraint-Induced Cognitive Therapy is a feasible and potentially effective cognitive rehabilitation approach for adults experiencing cognitive sequelae of long COVID. The intervention shows promise for enhancing performance in daily cognitive tasks and supporting return-to-work outcomes. For future clinical implementation, this approach may offer a structured and scalable option within multidisciplinary long COVID care. However, larger, well-powered studies are needed to confirm its efficacy and determine optimal delivery strategies across diverse populations and clinical settings. Future studies should include longitudinal occupational outcomes assessed over time, objective functional and cognitive assessments such as neuropsychological testing, pulmonary function tests, and neuroimaging, and larger samples enriched for working-age adults to better characterize the relationship between long COVID symptom clusters and return-to-work trajectories.

## 5. Conclusions

In this exploratory study of individuals who had COVID-19 infection, we preliminarily found three clusters of participants based on self-reported post-acute COVID symptoms. The ‘brain fog’ cluster identified a group of participants with symptoms related to cognitive performance which may be associated with work absence while the ‘undifferentiated’ cluster is a group of participants with no dominant symptoms, and the ‘persistent acute’ cluster is a group of participants with persistent, largely respiratory symptoms. These preliminary, hypothesis-generating findings suggest that long COVID, particularly with neurocognitive symptoms, may be associated with reduced workforce participation in some individuals, pending confirmation in larger, longitudinal studies.

## Supplementary Material

Supplemental table and figures

**Supplementary Materials:** The following supporting information can be downloaded at https://www.mdpi.com/article/10.3390/occuphealth1030031/s1. Figure S1: Elbow Plot for Optimal Number of Clusters; Figure S2: Silhouette plot for the K-means three-cluster solution; Table S1: Comparison of demographic and clinical characteristics between included (*n* = 273) and excluded (*n* = 274) participants.

## Figures and Tables

**Table 1.** Descriptive statistics of personal and clinical characteristics overall and by clusters.

Variables	Overall (*n* = 273)	Cluster 1 (*n* = 68)	Cluster 2 (*n* = 154)	Cluster 3 (*n* = 51)
**Age**, *Mean* (*SD*)	51.92 (14.02)	56.73 (12.34)	55.12 (14.45)	46.00 (9.90)
**Gender**				
Female	138 (50.5%)	39 (57.4%)	68 (44.2%)	31 (60.8%)
Male	91 (33.3%)	18 (26.5%)	53 (34.4%)	20 (39.2%)
Missing	44 (16.1%)	11 (16.2%)	33 (21.4%)	0 (0.0%)
**Race**				
Asian	9 (3.3%)	4 (5.9%)	5 (3.2%)	0 (0.0%)
Black or African American	52 (19.0%)	7 (10.3%)	37 (24.0%)	8 (15.7%)
Hispanic or Latino	1 (0.4%)	1 (1.5%)	0 (0.0%)	0 (0.0%)
White	159 (58.2%)	45 (66.2%)	73 (47.4%)	41 (80.4%)
Multiple	2 (0.7%)	0 (0.0%)	2 (1.3%)	0 (0.0%)
Decline/Refuse	1 (0.4%)	0 (0.0%)	1 (0.6%)	0 (0.0%)
Missing	49 (17.9%)	11 (16.2%)	36 (23.4%)	2 (3.9%)
**Hospitalization**				
No	171 (62.6%)	46 (67.6%)	79 (51.3%)	46 (90.2%)
Yes	58 (21.2%)	11 (16.2%)	42 (27.3%)	5 (9.8%)
Missing	44 (16.1%)	11 (16.2%)	33 (21.4%)	0 (0.0%)
**LOS (days)**, *Mean* (*SD*)	10.76 (14.02)	13.09 (11.12)	10.21 (15.06)	10.20 (12.24)
**Dx to Baseline (days)**, *Mean* (*SD*)	102.1 (141.00)	144.37 (160.01)	84.90 (135.17)	81.53 (114.33)

Abbreviations: SD; standard deviation. LOS; length of hospital stay. Dx to Baseline; days from COVID-19 diagnosis to baseline. Note. Percentages are based on the total number of participants within each cluster. Percentages may not sum to exactly 100% due to rounding.

**Table 2.** Descriptive statistics of post-acute COVID-19 symptoms (number of people who reported having the symptoms) overall and by clusters.

Variables	Overall (*n* = 273)	Cluster 1 (*n* = 68)	Cluster 2 (*n* = 154)	Cluster 3 (*n* = 51)
Don’t feel back to usual health	35 (12.8%)	15 (22.1%)	6 (3.9%)	14 (27.5%)
Fever	12 (4.4%)	0 (0.0%)	3 (1.9%)	9 (17.6%)
Felt Feverish	22 (8.1%)	1 (1.5%)	1 (0.6%)	20 (39.2%)
Chills	32 (11.7%)	6 (8.8%)	2 (1.3%)	24 (47.1%)
Muscle Aches	98 (35.9%)	42 (61.8%)	19 (12.3%)	37 (72.5%)
Runny Nose or Congestion	95 (34.8%)	33 (48.5%)	21 (13.6%)	41 (80.4%)
Sore Throat	31 (11.4%)	10 (14.7%)	3 (1.9%)	18 (35.3%)
Cough	94 (34.4%)	26 (38.2%)	21 (13.6%)	47 (92.2%)
Shortness of Breath (Dyspnea)	98 (35.9%)	53 (77.9%)	24 (15.6%)	21 (41.2%)
Nausea or Vomiting	27 (9.9%)	10 (14.7%)	1 (0.6%)	16 (31.4%)
Headaches	107 (39.2%)	47 (69.1%)	20 (13.0%)	40 (78.4%)
Abdominal Pain	33 (12.1%)	17 (25.0%)	6 (3.9%)	10 (19.6%)
Fatigue or Extreme Tiredness	155 (56.8%)	65 (95.6%)	44 (28.6%)	46 (90.2%)
Body Aches	119 (43.6%)	51 (75.0%)	28 (18.2%)	40 (78.4%)
Loss of Taste	65 (23.8%)	23 (33.8%)	12 (7.8%)	30 (58.8%)
Loss of Smell	67 (24.5%)	26 (38.2%)	11 (7.1%)	30 (58.8%)
Diarrhea	44 (16.1%)	17 (25.0%)	6 (3.9%)	21 (41.2%)
Confusion	60 (22.0%)	49 (72.1%)	6 (3.9%)	5 (9.8%)
Difficulty Concentrating	106 (38.8%)	64 (94.1%)	27 (17.5%)	15 (29.4%)
Difficulty Remembering Things	116 (42.5%)	66 (97.1%)	39 (25.3%)	11 (21.6%)
**Able to Carry Out Everyday Physical Activity**				
Completely	111 (40.7%)	8 (11.8%)	87 (56.5%)	16 (31.4%)
Mostly	55 (20.1%)	12 (17.6%)	35 (22.7%)	8 (15.7%)
Moderately	55 (20.1%)	20 (29.4%)	19 (12.3%)	16 (31.4%)
A Little	45 (16.5%)	25 (36.8%)	12 (7.8%)	8 (15.7%)
Not at All	7 (2.6%)	3 (4.4%)	1 (0.6%)	3 (5.9%)
**Bothered by Little Pleasure or Interest in Doing Things**				
Not at All	167 (61.2%)	12 (17.6%)	122 (79.2%)	33 (64.7%)
Several Days	57 (20.9%)	18 (26.5%)	29 (18.8%)	10 (19.6%)
More Than Half the Days	22 (8.1%)	14 (20.6%)	3 (1.9%)	5 (9.8%)
Nearly Everyday	27 (9.9%)	24 (35.3%)	0 (0.0%)	3 (5.9%)
**Bothered by Feeling Down, Depressed or Hopeless**				
Not at All	191 (70.0%)	16 (23.5%)	133 (86.4%)	42 (82.4%)
Several Days	56 (20.5%)	31 (45.6%)	19 (12.3%)	6 (11.8%)
More Than Half the Days	14 (5.1%)	11 (16.2%)	1 (0.6%)	2 (3.9%)
Nearly Everyday	12 (4.4%)	10 (14.7%)	1 (0.6%)	1 (2.0%)
**Anxiety Higher than Normal**				
Strongly Disagree	55 (20.1%)	3 (4.4%)	42 (27.3%)	10 (19.6%)
Disagree	64 (23.4%)	2 (2.9%)	49 (31.8%)	13 (25.5%)
Neutral	46 (16.8%)	10 (14.7%)	30 (19.5%)	6 (11.8%)
Agree	68 (24.9%)	30 (44.1%)	21 (13.6%)	17 (33.3%)
Strongly Agree	40 (14.7%)	23 (33.8%)	12 (7.8%)	5 (9.8%)

Note. Percentages are based on the total number of participants within each cluster. Percentages may not sum to exactly 100% due to rounding.

**Table 3.** Descriptive statistics of work status and the main reasons for not working (2-week follow-up) overall and by clusters.

Variables	Overall	Cluster 1	Cluster 2	Cluster 3
**Working for either pay or profit (*n* = 127)**				
Yes	75 (59.1%)	18 (56.3%)	45 (60.8%)	12 (57.1%)
No	52 (40.9%)	14 (43.8%)	29 (39.2%)	9 (42.9%)
**The Main Reasons for Not Working (*n* = 52)**				
I did not want to be employed at this time	2 (0.7%)	0 (0.0%)	2 (6.9%)	0 (0.0%)
I am/was sick with coronavirus symptoms	7 (2.6%)	7 (50.0%)	0 (0.0%)	0 (0.0%)
I am/was caring for an elderly person	1 (0.4%)	0 (0.0%)	1 (3.4%)	0 (0.0%)
I am/was sick (not coronavirus related) or disabled	8 (2.9%)	2 (14.3%)	6 (20.7%)	0 (0.0%)
I am retired	18 (6.6%)	1 (7.1%)	11 (37.9%)	6 (66.7%)
My employer experienced a reduction in business (Including furlough) due to the coronavirus pandemic	3 (1.1%)	1 (7.1%)	2 (6.9%)	0 (0.0%)
Other reason	13 (4.8%)	3 (21.4%)	7 (24.1%)	3 (33.3%)

Note. The working/not working percentages (yes/no) use as the denominator the number of participants within each cluster who responded to the 2-week follow-up survey (Cluster 1: *n* = 32; Cluster 2: *n* = 74; Cluster 3: *n* = 21), and that the reason-for-not-working percentages use the number of non-working respondents within each cluster as the denominator. Percentages may not sum to exactly 100% due to rounding.

## Data Availability

Data are available upon reasonable request to the corresponding author.

## References

[1] Mahase E. COVID-19: What do we know about “long covid”? BMJ 2020, 370, m2815. 32665317 10.1136/bmj.m2815

[2] Nabavi N. Long covid: How to define it and how to manage it. BMJ 2020, 370, m3489. 32895219 10.1136/bmj.m3489

[3] Wise J. Long covid: WHO calls on countries to offer patients more rehabilitation. BMJ 2021, 372, n405. 33568362 10.1136/bmj.n405

[4] National Academies of Sciences Engineering and Medicine. Long COVID: Examining Long-Term Health Effects of COVID-19 and Implications for the Social Security Administration; The National Academies Press: Washington, DC, USA, 2024. 36260712

[5] Fernández-de-las-Peñas C; Palacios-Ceña D; Gómez-Mayordomo V; Cuadrado ML; Florencio LL. Defining Post-COVID Symptoms (Post-Acute COVID, Long COVID, Persistent Post-COVID): An Integrative Classification. Int. J. Environ. Res. Public Health 2021, 18, 2621. 33807869 10.3390/ijerph18052621 PMC7967389

[6] Danesh V; Arroliga AC; Bourgeois JA; Boehm LM; McNeal MJ; Widmer AJ; McNeal TM; Kesler SR. Symptom clusters seen in adult COVID-19 recovery clinic care seekers. J. General. Intern. Med. 2023, 38, 442–449. 10.1007/s11606-022-07908-4 PMC9663188 36376627

[7] Frontera JA; Thorpe LE; Simon NM; de Havenon A; Yaghi S; Sabadia SB; Yang D; Lewis A; Melmed K; Balcer LJ; Post-acute sequelae of COVID-19 symptom phenotypes and therapeutic strategies: A prospective, observational study. PLoS ONE 2022, 17, e0275274. 36174032 10.1371/journal.pone.0275274 PMC9521913

[8] Reese JT; Blau H; Casiraghi E; Bergquist T; Loomba JJ; Callahan TJ; Laraway B; Antonescu C; Coleman B; Gargano M; Generalisable long COVID subtypes: Findings from the NIH N3C and RECOVER programmes. EBioMedicine 2023, 87, 1–17. 10.1016/j.ebiom.2022.104413 PMC9769411 36563487

[9] Thaweethai T; Jolley SE; Karlson EW; Levitan EB; Levy B; McComsey GA; McCorkell L; Nadkarni GN; Parthasarathy S; Singh U; Development of a definition of postacute sequelae of SARS-CoV-2 infection. JAMA 2023, 329, 1934–1946. 37278994 10.1001/jama.2023.8823 PMC10214179

[10] Davis HE; Assaf GS; McCorkell L; Wei H; Low RJ; Re’em Y; Redfield S; Austin JP; Akrami A. Characterizing long COVID in an international cohort: 7 months of symptoms and their impact. EClinicalMedicine 2021, 38, 101019. 34308300 10.1016/j.eclinm.2021.101019 PMC8280690

[11] U.S.C. Bureau. Measuring Household Experiences during the Coronavirus Pandemic. 2026. Available online: https://www.census.gov/data/experimental-data-products/household-pulse-survey.html (accessed on 26 December 2022).

[12] U.S.C. Bureau. Week 51 Household Pulse Survey: November 2–November 14. 2022. Available online: https://www.census.gov/data/tables/2022/demo/hhp/hhp51.html (accessed on 26 December 2022).

[13] Tenforde MW; Kim SS; Lindsell CJ; Rose EB; Shapiro NI; Files DC; Gibbs KW; Erickson HL; Steingrub JS; Smithline HA; Symptom duration and risk factors for delayed return to usual health among outpatients with COVID-19 in a multistate health care systems network—United States, March–June 2020. Morb. Mortal. Wkly. Rep. 2020, 69, 993–998. 10.15585/mmwr.mm6930e1 PMC7392393 32730238

[14] D’Souza G; Tong W; Gustafson D; Alcaide ML; Lahiri CD; Sharma A; French AL; Palella FJ; Kempf M-C; Mimiaga MJ; SARS-CoV-2 Infection Among People Living with HIV Compared with People Without HIV: Survey Results from the MACS-WIHS Combined Cohort Study. J. Acquir Immune Defic. Syndr. 2022, 89, 1–8. 34878431 10.1097/QAI.0000000000002822 PMC8667184

[15] Cirulli ET; Schiabor Barrett KM; Riffle S; Bolze A; Neveux I; Dabe S; Grzymski JJ; Lu JT; Washington NL. Long-term COVID-19 symptoms in a large unselected population. medRxiv 2020.

[16] Cook KF; Jensen SE; Schalet BD; Beaumont JL; Amtmann D; Czajkowski S; Dewalt DA; Fries JF; Pilkonis PA; Reeve BB; PROMIS measures of pain, fatigue, negative affect, physical function, and social function demonstrated clinical validity across a range of chronic conditions. J. Clin. Epidemiol. 2016, 73, 89–102. 26952842 10.1016/j.jclinepi.2015.08.038 PMC5131708

[17] Löwe B; Kroenke K; Gräfe K. Detecting and monitoring depression with a two-item questionnaire (PHQ-2). J. Psychosom. Res. 2005, 58, 163–171. 15820844 10.1016/j.jpsychores.2004.09.006

[18] R Core Team. Package ‘Cluster’. 2022. Available online: https://cran.r-project.org/web/packages/cluster/cluster.pdf (accessed on 17 March 2025).

[19] R Core Team. Package ‘Factoextra’. 2022. Available online: https://cran.r-project.org/web/packages/factoextra/factoextra.pdf (accessed on 7 March 2025).

[20] Cui M. Introduction to the k-means clustering algorithm based on the elbow method. Account. Audit. Financ. 2020, 1, 5–8.

[21] R Core Team. R: A Language and Environment for Statistical Computing. R Foundation for Statistical Computing. Available online: https://www.R-project.org/.

[22] Ziauddeen N; Gurdasani D; O’Hara ME; Hastie C; Roderick P; Yao G; Alwan NA. P108 Characteristics of long COVID: Findings from a social media survey. J. Epidemiol. Community Health 2021, 75, A90.

[23] Liviero F; Volpin A; Furlan P; Battistella M; Broggio A; Fabris L; Favretto F; Mason P; Cocchio S; Cozzolino C; The impact of SARS-CoV-2 on healthcare workers of a large University Hospital in the Veneto Region: Risk of infection and clinical presentation in relation to different pandemic phases and some relevant determinants. Front. Public Health 2023, 11, 1250911. 38098828 10.3389/fpubh.2023.1250911 PMC10720910

[24] Fernández-de-las-Peñas C; Martín-Guerrero JD; Florencio LL; Navarro-Pardo E; Rodríguez-Jiménez J; Torres-Macho J; Pellicer-Valero OJ. Clustering analysis reveals different profiles associating long-term post-COVID symptoms, COVID-19 symptoms at hospital admission and previous medical co-morbidities in previously hospitalized COVID-19 survivors. Infection 2023, 51, 61–69. 35451721 10.1007/s15010-022-01822-x PMC9028890

[25] Poletti S; Palladini M; Gennaro Mazza M; De Lorenzo R; The COVID-19 BioB Outpatient Clinic Study group; Furlan R; Ciceri F; Rovere-Querini P; Benedetti F. Long-term consequences of COVID-19 on cognitive functioning up to 6 months after discharge: Role of depression and impact on quality of life. Eur. Arch. Psychiatry Clin. Neurosci. 2022, 272, 773–782. 34698871 10.1007/s00406-021-01346-9 PMC8546751

[26] Tavares-Júnior JWL; de Souza ACC; Borges JWP; Oliveira DN; Siqueira-Neto JI; Sobreira-Neto MA; Braga-Neto P. COVID-19 associated cognitive impairment: A systematic review. Cortex 2022, 152, 77–79. 35537236 10.1016/j.cortex.2022.04.006 PMC9014565

[27] Uswatte G; Taub E; Ball K; Mitchell BS; Blake JA; McKay S; Biney F; Iosipchuk O; Hempfling P; Harris E; Long COVID brain fog treatment: An early-phase randomized controlled trial of constraint-induced cognitive therapy signals go. Rehabil. Psychol. 2026, 71, 46–61. 40310209 10.1037/rep0000626 PMC12323405

