# Supplementary material for: Post-Acute COVID-19 Symptoms Clusters and Work Status
Source: Occup Health (Auckl). Author manuscript; Available in PMC 2026 Aug 5. (PMC13431236; doi:10.3390/occuphealth1030031)
Supplement: Supplementary file 1 [file NIHMS2195808-supplement-Supplemental_table_and_figures.docx]

Table S1. Comparison of demographic and clinical characteristics between included (*n* = 273) and excluded (*n* = 274) participants.

|  | **Group** | | **Comparisons** | | |
| --- | --- | --- | --- | --- | --- |
|  | **Excluded** | **Included** | **q-value** | **Effect size** | **Interpretation** |
| Sample Size | 274 | 273 |  |  |  |
| **Age^a^** |  |  | 0.058 | 0.18 | Small Effect |
| Mean | 54.8 | 51.9 |  |  |  |
| SD | 14.3 | 14.0 |  |  |  |
| Median | 58.0 | 52.0 |  |  |  |
| IQR | 22.0 | 19.0 |  |  |  |
| Min | 22.0 | 18.0 |  |  |  |
| Max | 86.0 | 82.0 |  |  |  |
| **Gender ^b^** |  |  | 0.727 | 0.06 | Small Effect |
| F | 68 (24.8%) | 138 (50.5%) |  |  |  |
| M | 33 (12.0%) | 91 (33.3%) |  |  |  |
| Missing | 173 (63.1%) | 44 (16.1%) |  |  |  |
| **Race ^c^** |  |  |  |  |  |
| Asian | 1 (0.4%) | 9 (3.3%) | 0.181 | 0.15 | Small Effect |
| Black or African American | 34 (12.4%) | 52 (19.0%) |  |  |  |
| Decline/Refuse | 1 (0.4%) | 1 (0.4%) |  |  |  |
| Hispanic or Latino | 0 (0.0%) | 1 (0.4%) |  |  |  |
| Multiple | 1 (0.4%) | 2 (0.7%) |  |  |  |
| White | 60 (21.9%) | 159 (58.2%) |  |  |  |
| Missing | 177 (64.6%) | 49 (17.9%) |  |  |  |
| **Hospitalization ^b^** |  |  | 0.058 | 0.12 | Small Effect |
| No | 87 (31.8%) | 171 (62.6%) |  |  |  |
| Yes | 14 (5.1%) | 58 (21.2%) |  |  |  |
| Missing | 173 (63.1%) | 44 (16.1%) |  |  |  |

^a^ Mann-Whitney U and Rank-biserial correlation tests were conducted.

^b^ Chi-square and Cramér's V tests were conducted.

^c^ Fisher's exact and Cramér's V tests were conducted.

Figure S1. Elbow Plot for Optimal Number of Clusters.

Figure S2. Silhouette plot for the K-means three-cluster solution.
